# The Scleroderma Patient-centered Intervention Network Self-Management (SPIN-SELF) Program: protocol for a two-arm parallel partially nested randomized controlled feasibility trial with progression to full-scale trial

**DOI:** 10.1186/s13063-021-05827-z

**Published:** 2021-11-27

**Authors:** Julia Nordlund, Richard S. Henry, Linda Kwakkenbos, Marie-Eve Carrier, Brooke Levis, Warren R. Nielson, Susan J. Bartlett, Laura Dyas, Lydia Tao, Claire Fedoruk, Karen Nielsen, Marie Hudson, Janet Pope, Tracy Frech, Shadi Gholizadeh, Sindhu R. Johnson, Pamela Piotrowski, Lisa R. Jewett, Jessica Gordon, Lorinda Chung, Dan Bilsker, Alexander W. Levis, Kimberly A. Turner, Julie Cumin, Joep Welling, Catherine Fortuné, Catarina Leite, Karen Gottesman, Maureen Sauve, Tatiana Sofía Rodríguez-Reyna, Maggie Larche, Ward van Breda, Maria E. Suarez-Almazor, Amanda Wurz, Nicole Culos-Reed, Vanessa L. Malcarne, Maureen D. Mayes, Isabelle Boutron, Luc Mouthon, Andrea Benedetti, Brett D. Thombs, on behalf of the SPIN Investigators, Robert Riggs, Shervin Assassi, Ghassan El-Baalbaki, Carolyn Ells, Cornelia van den Ende, Kim Fligelstone, Amy Gietzen, Geneviève Guillot, Daphna Harel, Monique Hinchcliff, Christelle Nguyen, François Rannou, Michelle Richard, Anne A. Schouffoer, Christian Agard, Nassim Ait Abdallah, Alexandra Albert, Marc André, Elana J. Bernstein, Sabine Berthier, Lyne Bissonnette, Alessandra Bruns, Patricia Carreira, Marion Casadevall, Benjamin Chaigne, Chase Correia, Benjamin Crichi, Christopher Denton, Robyn Domsic, James V. Dunne, Bertrand Dunogue, Regina Fare, Dominique Farge-Bancel, Paul R. Fortin, Brigitte Granel-Rey, Genevieve Gyger, Eric Hachulla, Ariane L. Herrick, Sabrina Hoa, Alena Ikic, Niall Jones, Suzanne Kafaja, Nader Khalidi, Marc Lambert, David Launay, Hélène Maillard, Nancy Maltez, Joanne Manning, Isabelle Marie, Maria Martin, Thierry Martin, Ariel Masetto, François Maurier, Arsene Mekinian, Sheila Melchor, Mandana Nikpour, Louis Olagne, Vincent Poindron, Susanna Proudman, Alexis Régent, Sébastien Rivière, David Robinson, Esther Rodriguez, Sophie Roux, Perrine Smets, Vincent Sobanski, Robert Spiera, Virginia Steen, Evelyn Sutton, Carter Thorne, John Varga, Pearce Wilcox, Angelica Bourgeault, Mara Cañedo Ayala, Andrea Carboni Jiménez, Marie-Nicole Discepola, Maria Gagarine, Nora Østbø

**Affiliations:** 1 Lady Davis Institute of the Jewish General Hospital, 3755 Côte-Sainte-Catherine Road, Montreal, Quebec, H3T 1E2 Canada; 2 Department of Psychiatry, McGill University, Montreal, Quebec Canada; 3 Department of Clinical Psychology, Radboud University, Nijmegen, The Netherlands; 4 Department of Medical Psychology, Radboud Institute for Health Sciences, Radboud University Medical Center, Nijmegen, The Netherlands; 5 Centre for Prognosis Research, School of Medicine, Keele University, Keele, Staffordshire UK; 6 St. Joseph’s Health Care, London, Ontario Canada; 7 Department of Medicine, McGill University, Montreal, Quebec Canada; 8 Centre for Outcomes Research and Evaluation, Research Institute of the McGill University Health Centre, Montreal, Quebec Canada; 9 Scleroderma Foundation, Michigan Chapter, Southfield, MI USA; 10 Scleroderma Society of Ontario, Hamilton, Ontario Canada; 11 Department of Medicine, University of Western Ontario, London, Ontario Canada; 12 Department of Internal Medicine, University of Utah, Salt Lake City, UT USA; 13 California School of Professional Psychology/Alliant, Los Angeles, CA USA; 14 Toronto Scleroderma Program, Mount Sinai Hospital & Toronto Western Hospital, Toronto, Ontario Canada; 15 Institute of Health Policy, Management, and Evaluation, University of Toronto, Toronto, Ontario Canada; 16 Private practice – Nutrition, Milton, Ontario Canada; 17 Department of Psychology, Jewish General Hospital, Montreal, Quebec Canada; 18 Department of Medicine, Hospital for Special Surgery, New York City, NY USA; 19 Department of Medicine, Stanford University, Palo Alto, CA USA; 20 Department of Medicine, Palo Alto VA Health Care System, Palo Alto, CA USA; 21 Faculty of Health Sciences, Simon Fraser University, Burnaby, British Columbia Canada; 22 Department of Psychiatry, University of British Columbia, Vancouver, British Columbia Canada; 23 Department of Biostatistics, Harvard T. H. Chan School of Public Health, Boston, MA USA; 24 NVLE Dutch patient organization for systemic autoimmune diseases, Utrecht, The Netherlands; 25 Ottawa Scleroderma Support Group, Ottawa, Ontario Canada; 26 University of Minho, Braga, Portugal; 27 Scleroderma Foundation, Los Angeles, CA USA; 28 Scleroderma Canada, Hamilton, Ontario Canada; 29 Instituto Nacional de Ciencias Médicas y Nutrición Salvador Zubirán, Mexico City, Mexico; 30 Department of Health Research Methods, Evidence and Impact, McMaster University, Hamilton, Ontario Canada; 31 Faculty of Behavioural and Movement Sciences, VU University, Amsterdam, The Netherlands; 32 Department of General Internal Medicine, University of Texas MD Anderson Cancer Center, Houston, TX USA; 33 School of Kinesiology, University of the Fraser Valley, Chilliwack, British Columbia Canada; 34 Faculty of Kinesiology, University of Calgary, Calgary, Alberta Canada; 35 Department of Oncology, Cumming School of Medicine, Calgary, Canada; 36 Department of Psychosocial Resources, Tom Baker Cancer Centre, Alberta Health Services, Calgary, Alberta Canada; 37 Department of Psychology, San Diego State University, San Diego, CA USA; 38 Joint Doctoral Program in Clinical Psychology, San Diego State University/University of California San Diego, San Diego, CA USA; 39 Department of Internal Medicine, University of Texas McGovern School of Medicine, Houston, TX USA; 40 Université de Paris, Centre of Research Epidemiology and Statistics (CRESS), Inserm, INRA, Paris, France; 41 Centre d’Épidémiologie Clinique, Assistance Publique–Hôpitaux de Paris (AP-HP), Hôpital Hôtel Dieu, Paris, France; 42 Service de Médecine Interne, Centre de Référence Maladies Autoimmunes Systémiques Rares d’Ile de France, Hôpital Cochin, Assistance Publique-Hôpitaux de Paris (AP-HP), Paris, France; 43 APHP-CUP, Hôpital Cochin, Université de Paris, F-75014 Paris, France; 44 Department of Epidemiology, Biostatistics, and Occupational Health, McGill University, Montreal, Quebec Canada; 45 Respiratory Epidemiology and Clinical Research Unit, McGill University Health Centre, Montreal, Quebec Canada; 46 Department of Psychology, McGill University, Montreal, Quebec Canada; 47 Department of Educational and Counselling Psychology, McGill University, Montreal, Quebec Canada; 48 Biomedical Ethics Unit, McGill University, Montreal, Quebec Canada

**Keywords:** Scleroderma, Systemic sclerosis, Self-management, Self-efficacy, Patient activation, Randomized controlled trial, e-Health

## Abstract

**Background:**

Systemic sclerosis (scleroderma; SSc) is a rare autoimmune connective tissue disease. We completed an initial feasibility trial of an online self-administered version of the Scleroderma Patient-centered Intervention Network Self-Management (SPIN-SELF) Program using the cohort multiple randomized controlled trial (RCT) design. Due to low intervention offer uptake, we will conduct a new feasibility trial with progression to full-scale trial, using a two-arm parallel, partially nested RCT design. The SPIN-SELF Program has also been revised to include facilitator-led videoconference group sessions in addition to online material. We will test the group-based intervention delivery format, then evaluate the effect of the SPIN-SELF Program on disease management self-efficacy (primary) and patient activation, social appearance anxiety, and functional health outcomes (secondary).

**Methods:**

This study is a feasibility trial with progression to full-scale RCT, pending meeting pre-defined criteria, of the SPIN-SELF Program. Participants will be recruited from the ongoing SPIN Cohort (http://www.spinsclero.com/en/cohort) and via social media and partner patient organizations. Eligible participants must have SSc and low to moderate disease management self-efficacy (Self-Efficacy for Managing Chronic Disease (SEMCD) Scale score ≤ 7.0). Participants will be randomized (1:1 allocation) to the group-based SPIN-SELF Program or usual care for 3 months. The primary outcome in the full-scale trial will be disease management self-efficacy based on SEMCD Scale scores at 3 months post-randomization. Secondary outcomes include SEMCD scores 6 months post-randomization plus patient activation, social appearance anxiety, and functional health outcomes at 3 and 6 months post-randomization. We will include 40 participants to assess feasibility. At the end of the feasibility portion, stoppage criteria will be used to determine if the trial procedures or SPIN-SELF Program need important modifications, thereby requiring a re-set for the full-scale trial. Otherwise, the full-scale RCT will proceed, and outcome data from the feasibility portion will be utilized in the full-scale trial. In the full-scale RCT, 524 participants will be recruited.

**Discussion:**

The SPIN-SELF Program may improve disease management self-efficacy, patient activation, social appearance anxiety, and functional health outcomes in people with SSc. SPIN works with partner patient organizations around the world to disseminate its programs free-of-charge.

**Trial registration:**

ClinicalTrials.govNCT04246528. Registered on 27 January 2020

**Supplementary Information:**

The online version contains supplementary material available at 10.1186/s13063-021-05827-z.

## Introduction

Rare diseases, which together affect an estimated 6–8% of people worldwide, are defined as conditions with a prevalence rate fewer than 1 in 2000 [1]. Systemic sclerosis (scleroderma (SSc)) is a rare, chronic, autoimmune disease characterized by abnormal fibrotic processes. It can affect multiple organ systems, including the skin, lungs, kidneys, and gastrointestinal tract. Onset most commonly occurs between ages 30 and 50 years, and approximately 80% of people with SSc are women [2, 3]. Common manifestations include Raynaud’s phenomenon, skin thickening, dyspnea and cough, gastroesophageal reflux, and other gastrointestinal symptoms [2, 3]. Problems that impact daily life include mobility limitations, pain, fatigue, pruritus, sleep difficulty, depressive symptoms, and body image concerns from changes in appearance (e.g., skin tightening, pigmentation, telangiectasias) [4–9]. There is currently no cure and treatment is complex, typically involving multiple healthcare professionals [10].

Chronic disease self-management involves active participation in one’s health care to better manage symptoms and improve coping. Self-management programs have been shown to be effective and are widely disseminated in many chronic diseases [11–13]. There are, however, barriers to developing, testing, and disseminating self-management programs in rare diseases, including SSc. People with SSc face unique challenges that are not addressed by generic self-management programs [14, 15]. Additionally, in a rare disease context, it is difficult to recruit a sufficient number of participants to conduct robust clinical trials and disseminate effective interventions [14]. The only randomized controlled trial (RCT) of a self-management intervention in SSc compared a self-administered internet-based program to an educational book among 267 people with SSc from the USA and found that the primary outcome, disease-management self-efficacy, and secondary outcomes were not improved [16].

The Scleroderma Patient-centered Intervention Network (SPIN) [17, 18] is an international collaboration of SSc researchers, health care providers, patient organizations, and people with SSc. SPIN maintains a large multinational SSc cohort (http://www.spinsclero.com/en/cohort) with over 1600 active participants from 47 centers in 7 countries to collect longitudinal data on patient-reported outcomes and as a framework for embedding RCTs of educational, rehabilitation, psychological, and self-management interventions [17, 18]. The SPIN Self-Management (SPIN-SELF) Program was designed based on successful self-management programs for more common diseases [11–13] and informed by research on concerns and coping challenges of people with SSc, input from people with SSc and health care professionals who participated in a series of focus groups [15], and input from SPIN’s Patient Advisory Board. The program includes 9 online modules, available in English and French.

We conducted an initial feasibility trial of a self-administered online version of the SPIN-SELF Program [19, 20] using the cohort multiple randomized controlled trial (cmRCT) design [21–23], a randomized consent design [24] in which eligible participants, based on their cohort assessments, are randomized to be offered an intervention or to a usual care control prior to being told about the trial. Among 40 participants in the feasibility trial, 26 were offered to try the program (3:2 allocation) and only 9 (35%) consented to use the program. Program usage was low; 2 of 9 users (22%) logged into the program once, and 7 of 9 (78%) did not access any module [20]. There are few published RCTs that have used the cmRCT design, and intervention offer acceptance has been low (40–60%) in these trials [22, 23], as it was in a recently completed SPIN trial of an online hand exercises program (*N* = 466; 61%) [25].

Thus, we have made two major revisions for the present feasibility trial with progression to full-scale trial to address the low acceptance of offer to try the intervention from the initial feasibility trial. First, instead of the cmRCT design, we will obtain consent for the trial and randomize post-consent to the SPIN-SELF Program or usual care. Second, we will incorporate 8 videoconference-based groups led by peer facilitators to support delivery of the online program material, similar to what we did in our recent successful trial of an intervention to address mental health symptoms in the early stage of COVID-19 [26, 27].

To test our revised trial design and program delivery method, we will conduct a new feasibility trial with direct progression to full-scale trial [28, 29] if certain criteria are met. The feasibility trial will determine whether, prior to proceeding to the planned full-scale RCT, (1) further adaptations are needed to the research design and trial procedures or (2) improvements are needed to the group delivery format. We plan to progress directly from the feasibility trial to the full-scale trial, including results from the feasibility trial in the full-scale RCT, unless one or more pre-defined stoppage criteria occur. If stoppage criteria occur, we will address identified issues and begin the full-scale trial anew, separate from the feasibility trial. In the planned full-scale RCT, we will evaluate the effect of the SPIN-SELF Program, compared to usual care, on disease management self-efficacy 3 months post-randomization (primary outcome) and on self-efficacy 6 months post-randomization and patient activation, social appearance anxiety, and functional health outcomes 3 and 6 months post-randomization (secondary outcomes). We selected a usual care comparison group since this is a pragmatic trial intended to test the effect of adding the self-management program to usual care.

## Methods

The SPIN-SELF feasibility trial with progression to full-scale trial will be a pragmatic, two-arm, parallel, partially nested RCT (PN-RCT) [30] with 1:1 randomization to intervention or usual care. We will recruit participants from the ongoing SPIN Cohort and externally via social media and patient organization partners. We will use the PN-RCT design because participants randomized to the intervention arm will be clustered into intervention groups, whereas usual care control participants will not be clustered.

The trial has been registered (ClinicalTrials.gov, NCT04246528), and the protocol follows Standard Protocol Items Recommendations for Interventional Trials (SPIRIT) 2013 Statement reporting recommendations [31]. Reporting was also informed by Consolidated Standard of Reporting Trials statement extensions for nonpharmacologic trials [32] pragmatic trials [33], and trials conducted using cohorts and routinely collected data [34]. All items from the World Health Organization trial registration data set are available as Additional file 1, and the SPIRIT checklist of recommended items to address in a clinical trial protocol is available as Additional file 2. The participant consent form is provided in Additional file 3.

**Fig. 1:**
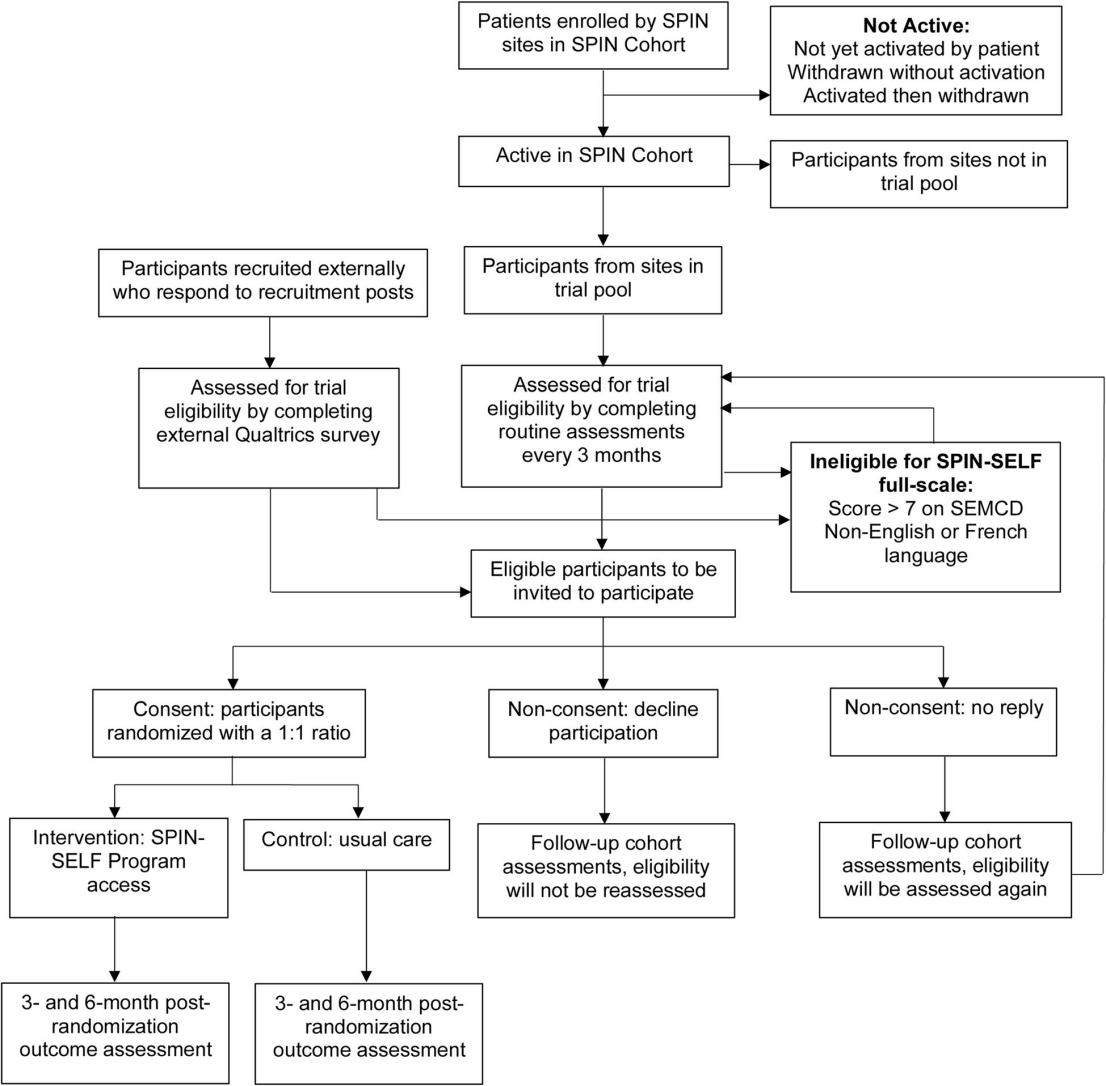
SPIN Cohort and non-SPIN Cohort participants: trial flow diagram

**Fig. 2:**
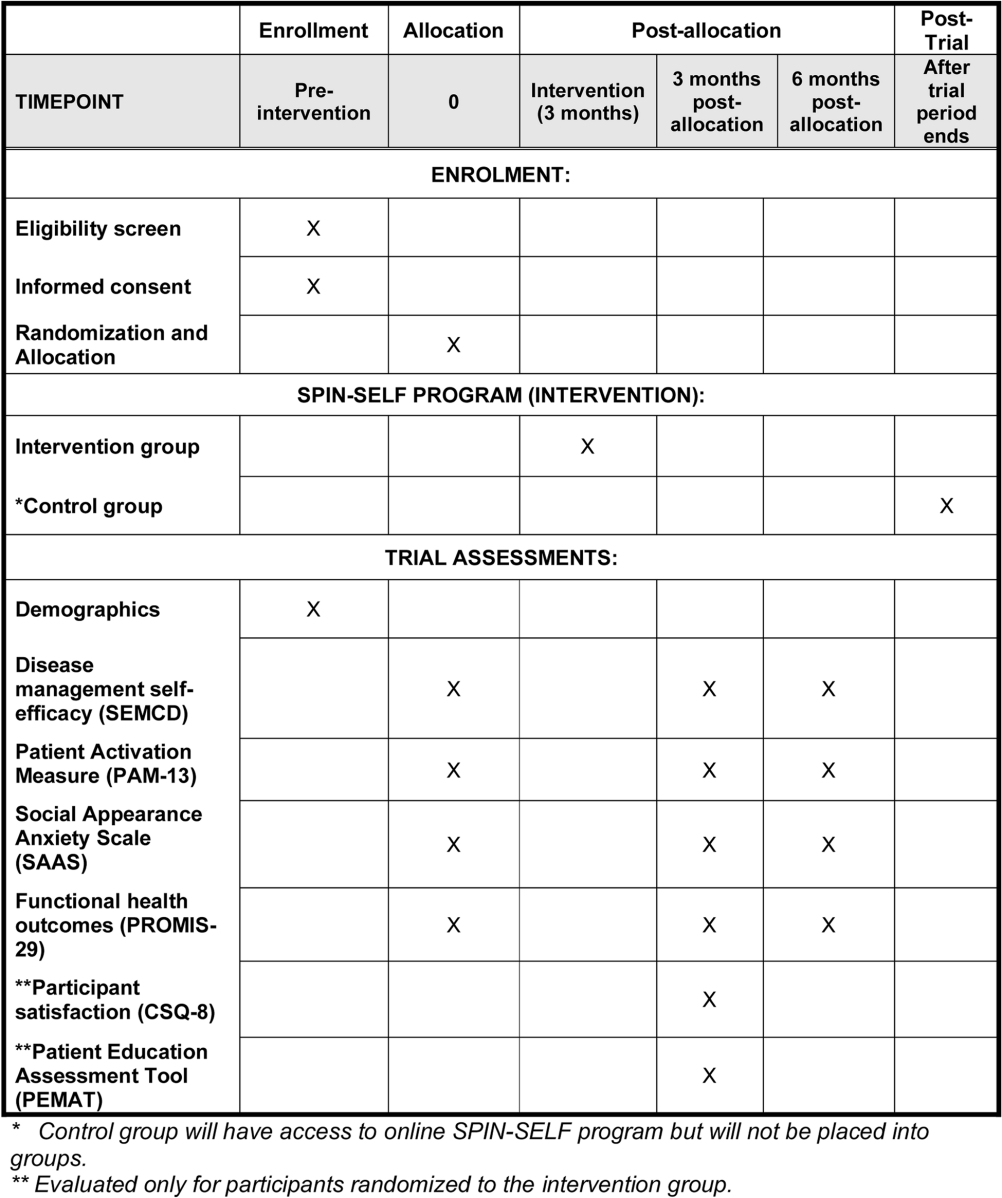
Schedule of enrollment, interventions, and assessments

Figure 1 provides the planned flow of participants and Fig. 2 the planned schedule of enrollment, intervention, and assessments.

### Progression from SPIN-SELF feasibility to full-scale trial

During the feasibility portion of the trial, we will collect information on (1) trial procedures that may help us to optimize trial management for the full-scale trial but would not lead to stoppage and re-start for the full-scale trial and (2) stoppage criteria that will determine if we can proceed directly from the feasibility trial to the full-scale trial or if substantial revisions require starting the full-scale trial anew. See Table 1.

**Table 1:** Feasibility elements of trial procedures and stoppage criteria

Feasibility elements to be evaluated to improve for full-scale trial^**a**^	
Trial procedure	Data to be collected
Recruitment and enrollment procedures	Information on eligible SPIN Cohort participants who consent to be enrolled need to be extracted from the SPIN Cohort platform and integrated with information from participants recruited externally and who enroll via a *Qualtrics* survey into an Excel tracking sheet. We will evaluate procedure efficiency and accuracy.
Proportion of eligible participants who consent to participate in trial	In cases where patients who are eligible for SPIN-SELF decline to participate in the feasibility trial, the trial team will collect information on why they declined via *Qualtrics*, including suggestions to improve recruitment or enrollment procedures.
Contact protocol adherence	Participants who consent to enroll will be contacted by a member of the SPIN research team to confirm their availability, and answer their questions. All contacts with participants will be tracked. We will evaluate adherence to the contact protocol and whether procedures can be improved.
Personnel requirements to enroll participants, support facilitators, and help participants accessing the different platforms used (*Qualtrics*, GoToMeeting, SPIN-SELF)	Should there be barriers in these procedures, personnel input will be collected to identify ways to improve them.
Intervention enrollment procedure	We will provide intervention group participants access to the SPIN-SELF online program by enrolling them via a user registration platform. This platform will ensure that only intervention participants are granted access to the program. We will conduct an audit of registered users with access and compare to allocation lists every 3 days (during enrollment), and we will evaluate our procedures if errors occur.
Proportion of eligible patients who consent to participate in the feasibility trial	If fewer than 50% of eligible patients consent to participate in the feasibility trial, the full-scale trial may proceed but barriers will need to be identified to improve participation. Participant feedback will be used to modify recruitment and enrollment procedures accordingly.
Fidelity of the delivery of the intervention (i.e., group sessions)	We will evaluate fidelity to group session protocols using a pre-established checklist. If sessions are not delivered according to the pre-specified program, the trial team will investigate to identify barriers to the delivery of the intervention and will modify the intervention procedures accordingly.
Group format of the SPIN-SELF sessions	We will collect feedback from intervention group participants on the format of the intervention (via the CSQ-8 and PEMAT questionnaires). We will also contact trial participants who miss 2 or more group sessions, to inquire why they missed those sessions and ask them what about the trial procedures could have been improved to ensure their attendance.
**Stoppage criteria**^**b**^	
**Trial procedures**	**Criteria**
Recruitment and enrollment of trial participants	If participants who are eligible based on their self-efficacy scores (SEMCD ≤ 7.0) are not recorded as eligible in the SPIN Cohort platform and not recruited, then the programming errors in the platform will need to be addressed and procedures will need to be re-set before the full-scale trial can commence. If more than 6 non-eligible participants (15% of 40 enrolled) are erroneously identified as eligible and assigned to the intervention or waitlist control, we will address programming errors, modify our procedures and conduct the full-scale trial as a new trial.
Fidelity of the delivery of the intervention (i.e., group sessions)	If by the end of the feasibility trial > 20% of the components of the 8 sessions were not delivered according to the pre-specified program, we will investigate to identify barriers to the delivery of the intervention and will modify the intervention procedures accordingly, before proceeding to the full-scale trial. The full-scale trial would need to commence as a new trial.
Group format of the SPIN-SELF sessions	If participants across groups consistently suggest similar changes to the format of the SPIN-SELF Program, then we will modify the format of the intervention sessions according to this consensus feedback. These modifications would be made before commencing the full-scale trial as a new trial.

^a^We have identified specific trial procedures for which we will collect information to optimize or improve them and further ensure their success and efficiency

^b^We have identified stoppage criteria which will be used to assess whether the full-scale trial can proceed with or without modifications to the procedures by evaluating whether certain targets are met. Because we have previously tested many of these procedures and because funding is in place for the full-scale trial, relatively few stoppage criteria have been identified. Once this evaluation is complete, the trial team will decide which of the following scenarios is most appropriate: (A) Proceed to the full-scale trial without modifications to the trial procedures or intervention delivery, (B) make minor modifications to trial procedures that do not affect participant experience meaningfully and continue to the full-scale trial, or (C) make important modifications to trial procedures or intervention delivery and start full-scale trial as a new trial with modifications in place. The outcome data from the internal feasibility trial will be utilized in the analyses of the full-scale trial in scenarios A and B but not C. In the table, we have identified scenarios that may require modifications to trial procedures and lead to the decision to conduct the full-scale trial as a new trial post-modification (scenario C)

Many of the trial management procedures to be used in the SPIN-SELF trial have been successfully tested in previous SPIN trials with common design and intervention delivery features, including the SPIN Scleroderma Support Group Leader Education Program (SPIN-SSLED) Trial [35, 36], SPIN COVID-19 Home-isolation Activities Together Program (SPIN-CHAT) Trial [26, 27], SPIN Hand Function Program (SPIN-HAND) Trial [25], and initial SPIN-SELF Feasibility Trial [20] and including procedures for recruitment, enrollment, randomization, and delivery of an online intervention, which do not need additional feasibility verification. Thus, as shown in Table 1, we have identified only 3 stoppage criteria. Based on evaluation of feasibility aspects and stoppage criteria, we will either (A) progress to the full-scale trial without modifications to the trial procedures or intervention delivery, (B) make minor modifications to trial procedures that do not affect participant experience meaningfully and progress to the full-scale trial, or (C) make important modifications to trial procedures or intervention delivery and start the full-scale trial as a new trial with modifications in place. The outcome data from the proposed internal feasibility trial will be utilized in the analyses of the full-scale trial in scenarios A and B but not C.

### SPIN Cohort participants

To be eligible for the SPIN Cohort, participants must meet 2013 American College of Rheumatology / European League Against Rheumatism SSc classification criteria [37]; be ≥18 years old; be fluent in English, French, or Spanish; and be able to respond to questionnaires via the internet. The SPIN Cohort is a convenience sample. Eligible SPIN Cohort participants are recruited and provide written consent at one of one of 47 SPIN sites [18] from Canada, the USA, the UK, France, Spain, Mexico, and Australia during regular medical visits. Site personnel submit an online medical data form to enroll participants, after which participants receive an automated email with instructions to activate their account and complete online SPIN Cohort measures. Cohort participants complete outcome measures via the Internet upon enrollment and subsequently every 3 months. Participants consent to allow their data to be used for observational studies and be contacted by SPIN personnel about other SPIN studies. To date, over 2000 people have enrolled (1615 active) [18].

### SPIN-SELF trial participants

We plan to enroll 40 English-speaking participants in the feasibility portion of the trial and a total of 524 English- and French-speaking participants in the full-scale trial (including feasibility participants if progression occurs). Eligible participants must (1) meet 2013 American College of Rheumatology / European League Against Rheumatism SSc classification criteria [37], verified via SPIN Cohort enrollment or self-report by external trial participants that they were classified as having SSc by a rheumatologist or internist; (2) have low to moderate disease management self-efficacy (Self-Efficacy for Managing Chronic Disease (SEMCD) Scale [38] ≤ 7.0); and (3) not have been enrolled and assigned to the intervention arm of the initial SPIN-SELF Feasibility Trial [20] (control participants were not notified about the trial and had outcomes assessed via regular cohort assessments). Due to the smaller number of French-speaking participants in the SPIN Cohort, only English-speaking participants will be included in the feasibility portion of the trial to ensure there will be an adequate number of French-speaking participants in the full-scale RCT.

### Intervention and comparator

**Fig. 3:**
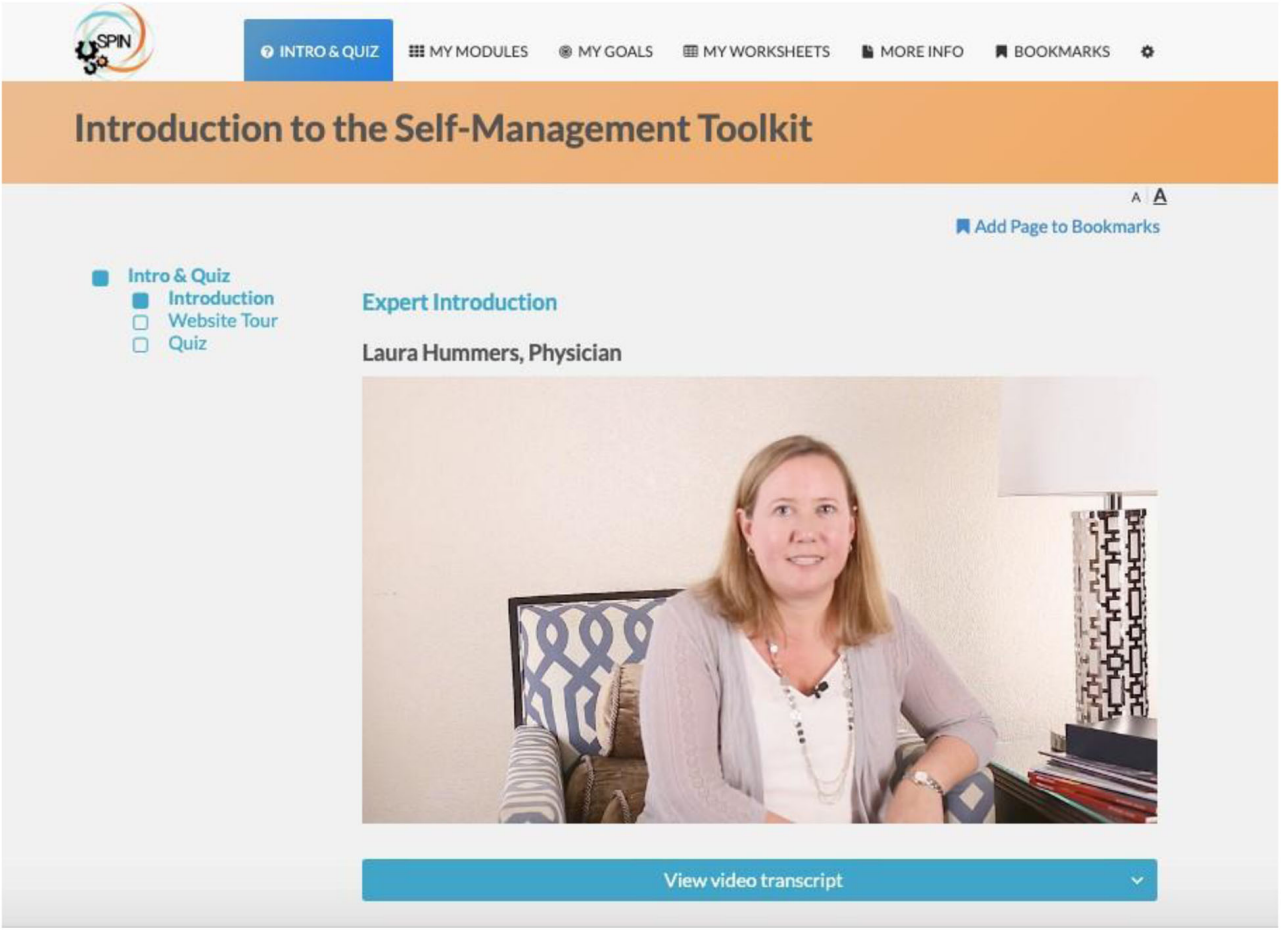
Health care professional discussing the SPIN-SELF Program

**Fig. 4:**
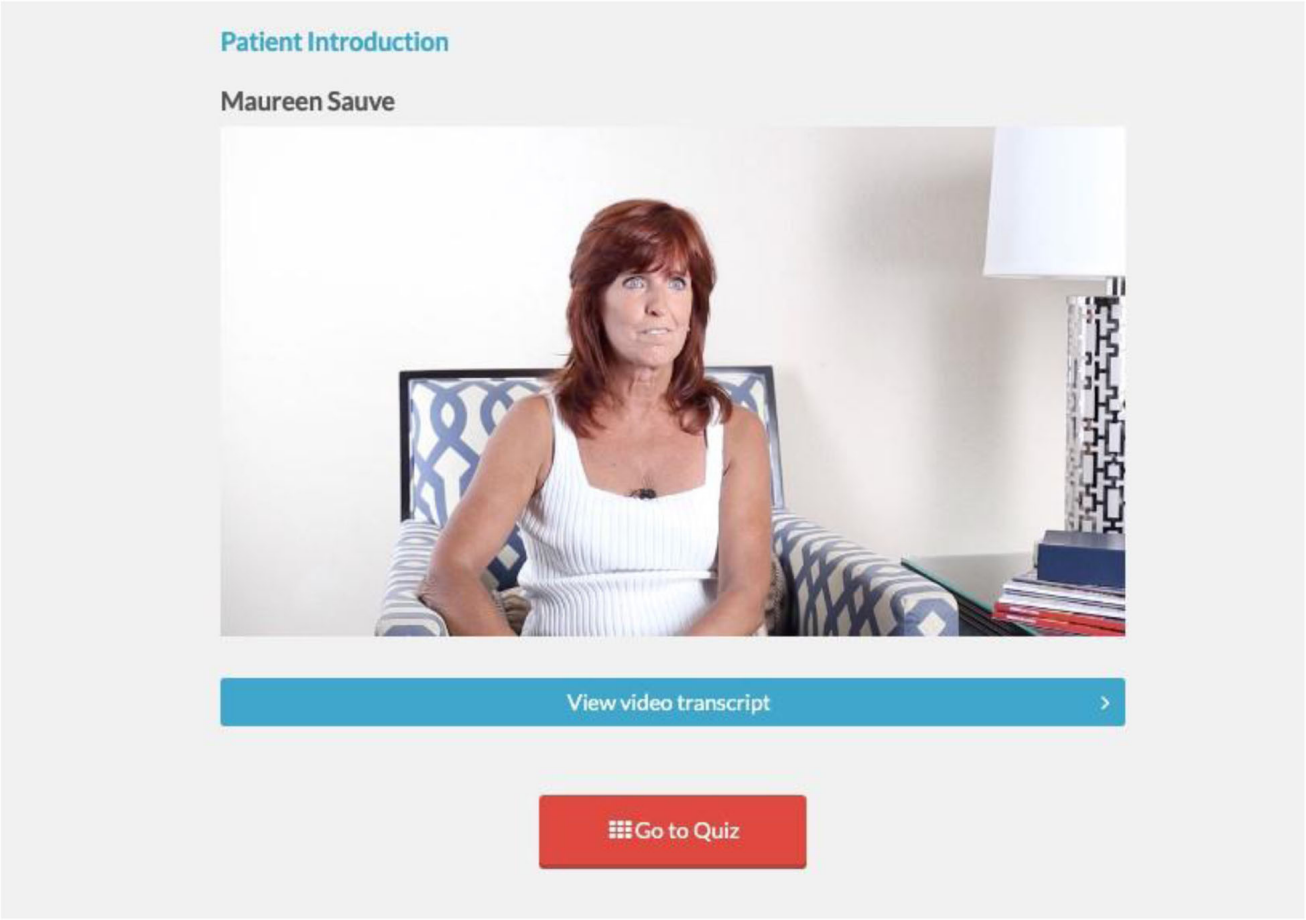
Person with SSc discussing their experience with self-management

**Fig. 5:**
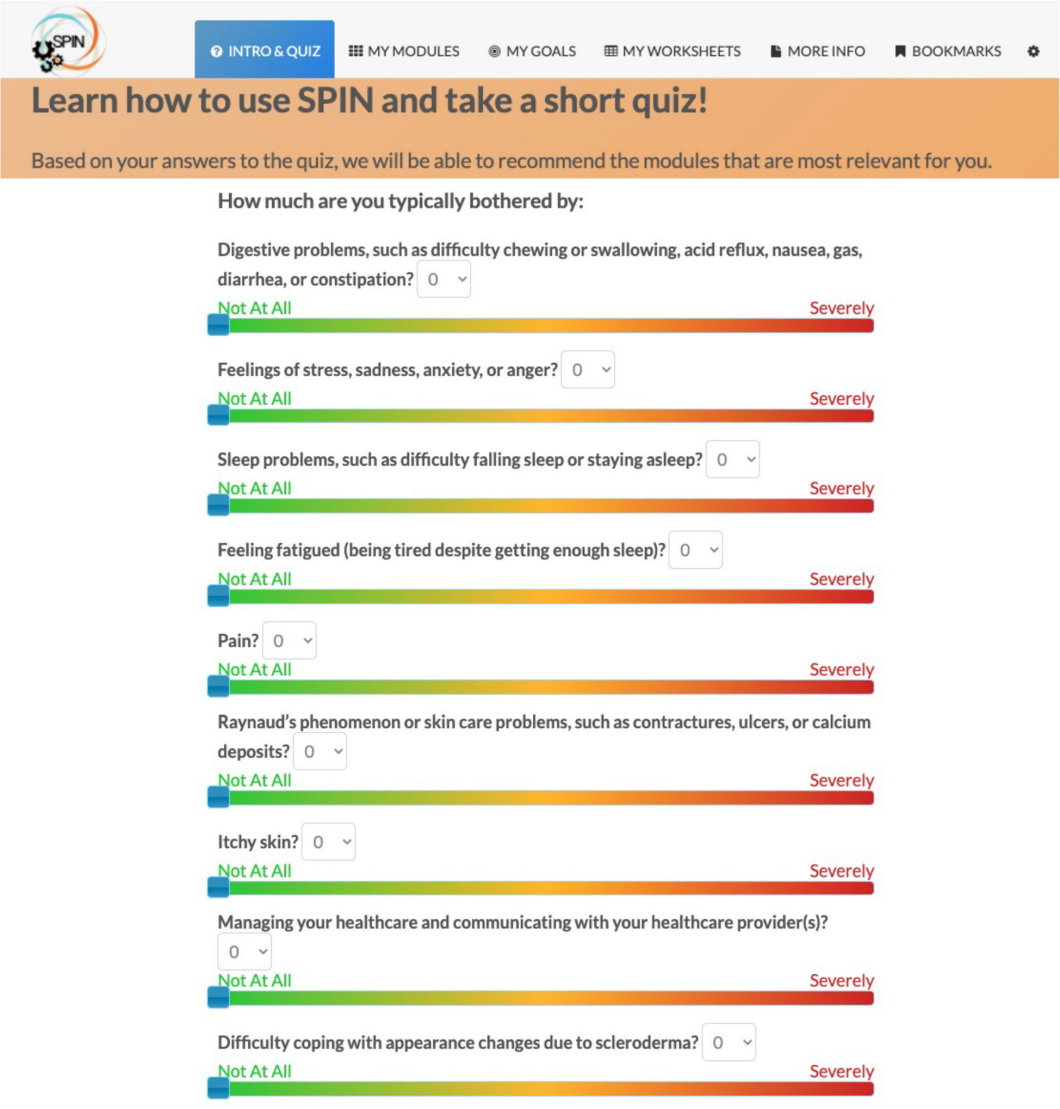
Nine-item quiz providing guidance to modules most relevant to participants’ symptoms and disease management challenges

**Fig. 6:**
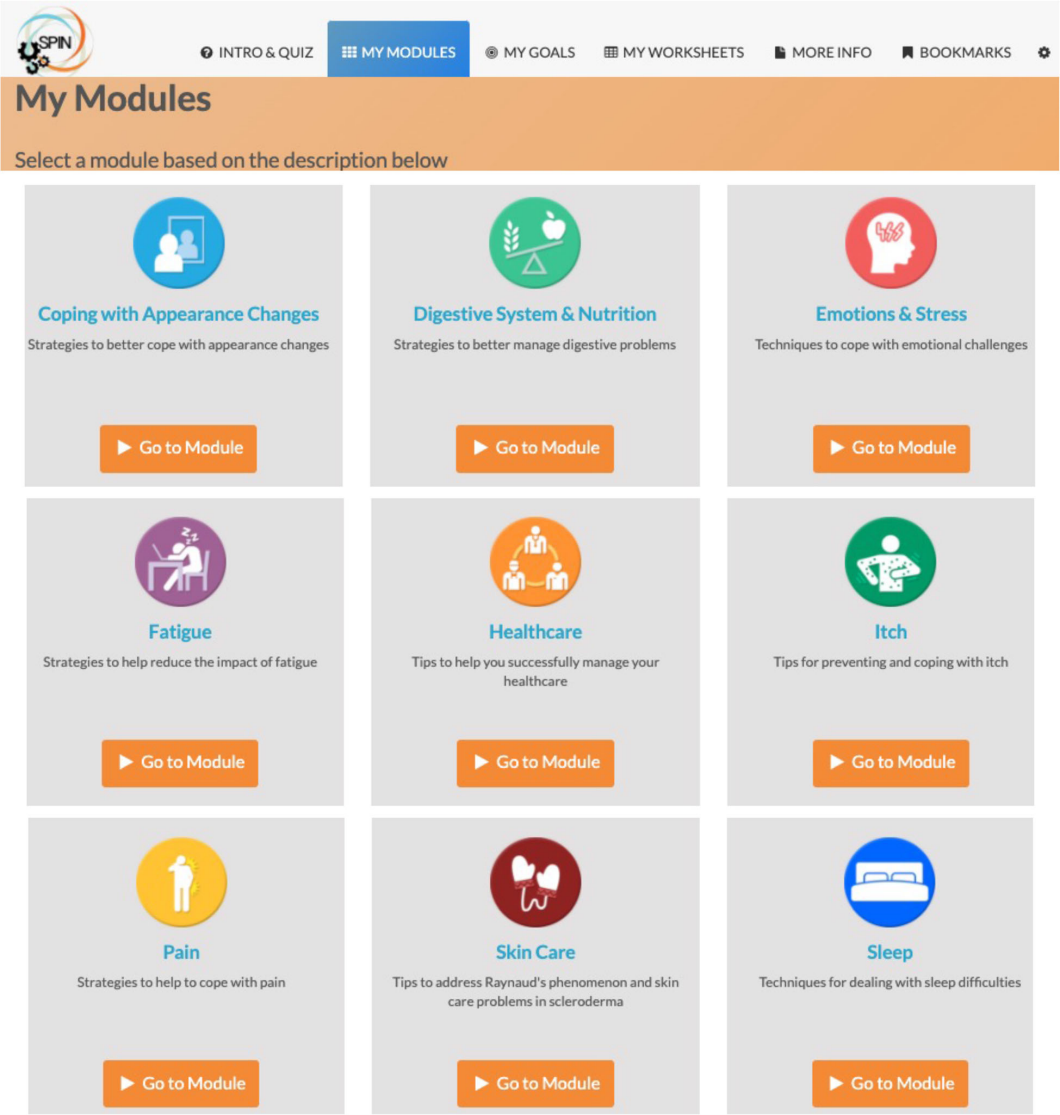
Menu of the SPIN-SELF Program modules

**Fig. 7:**
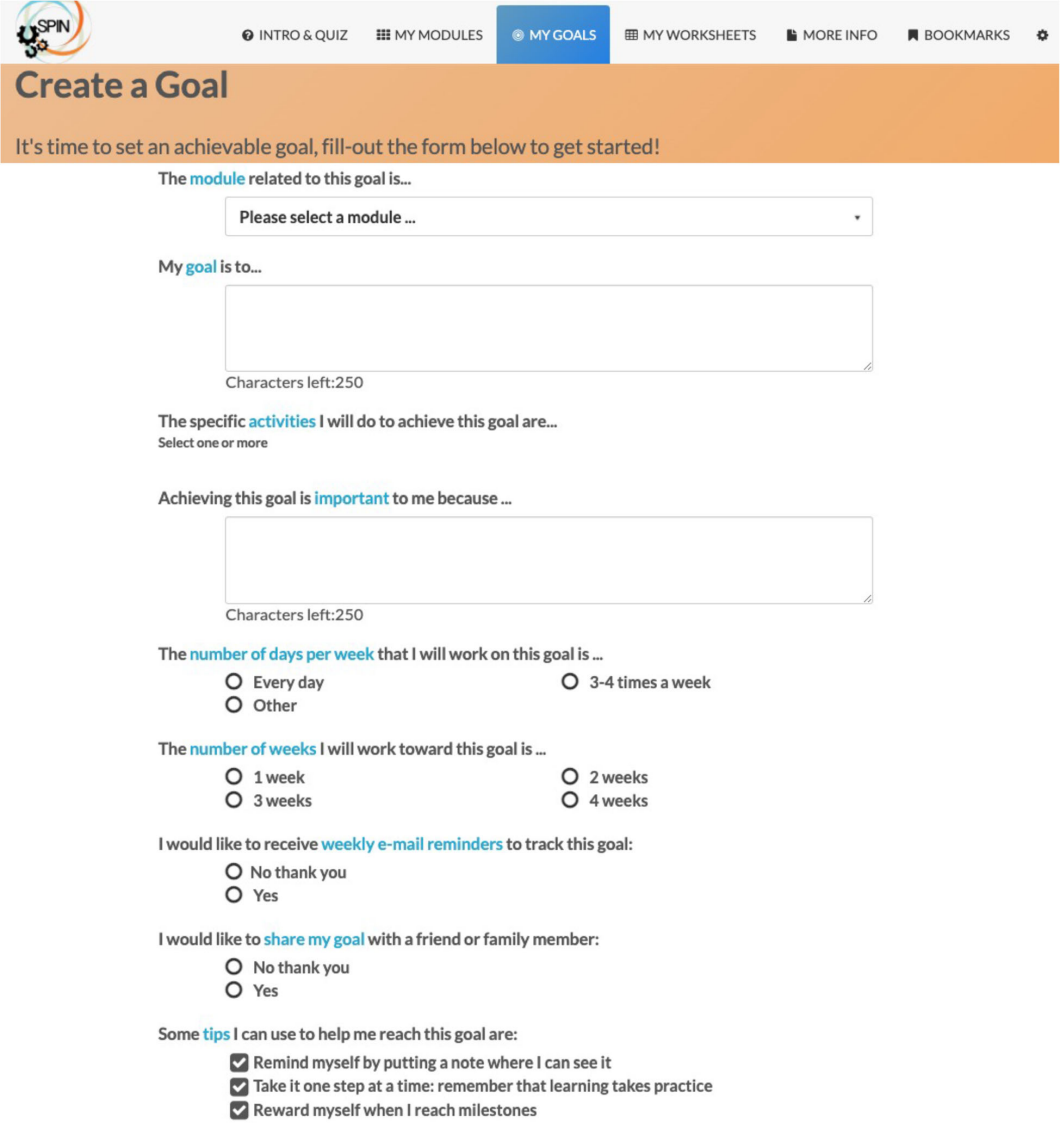
Example of the goal-setting feature in the SPIN-SELF Program

**Fig. 8:**
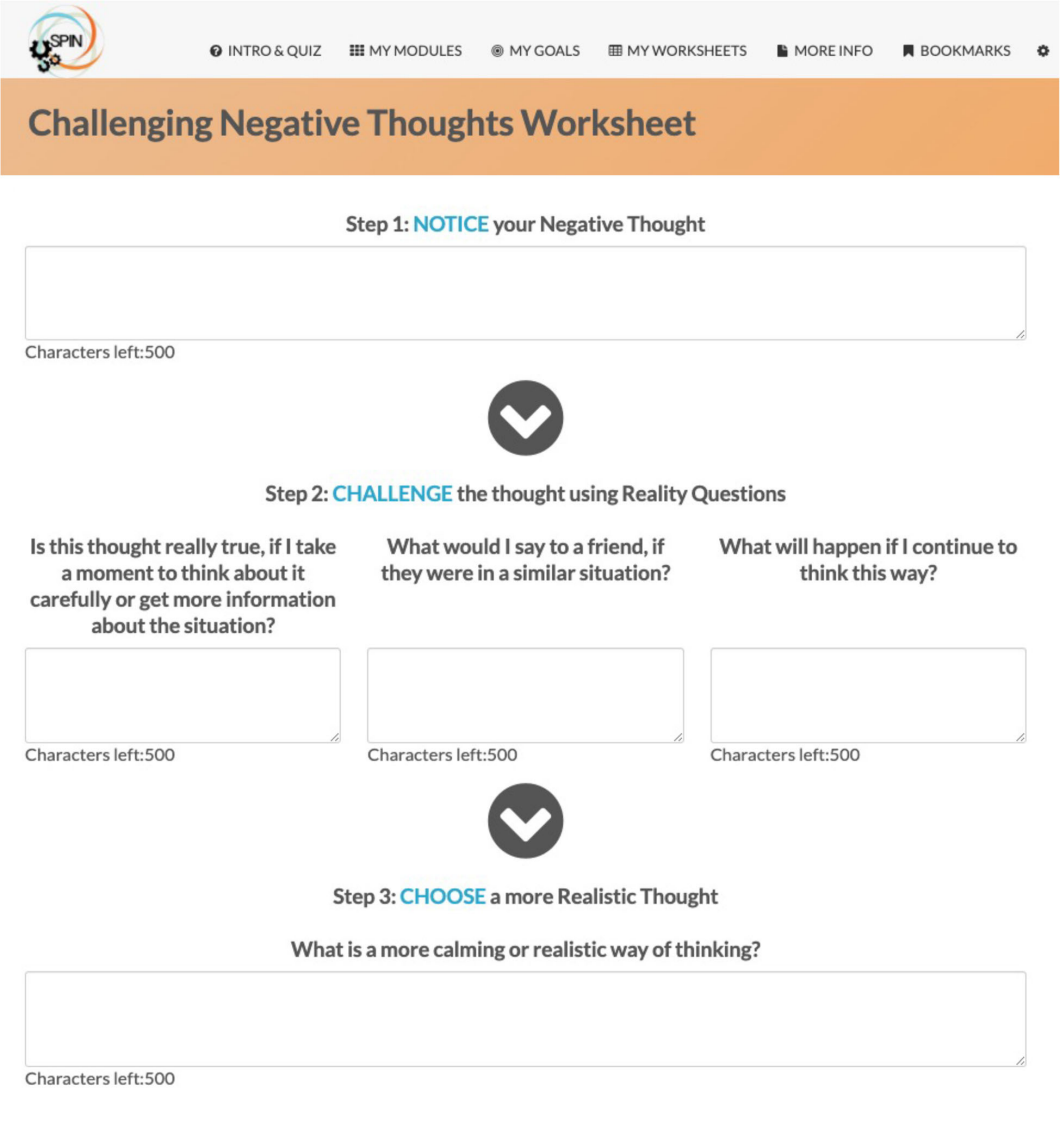
Example of the worksheet features in the SPIN-SELF Program

**Fig. 9:**
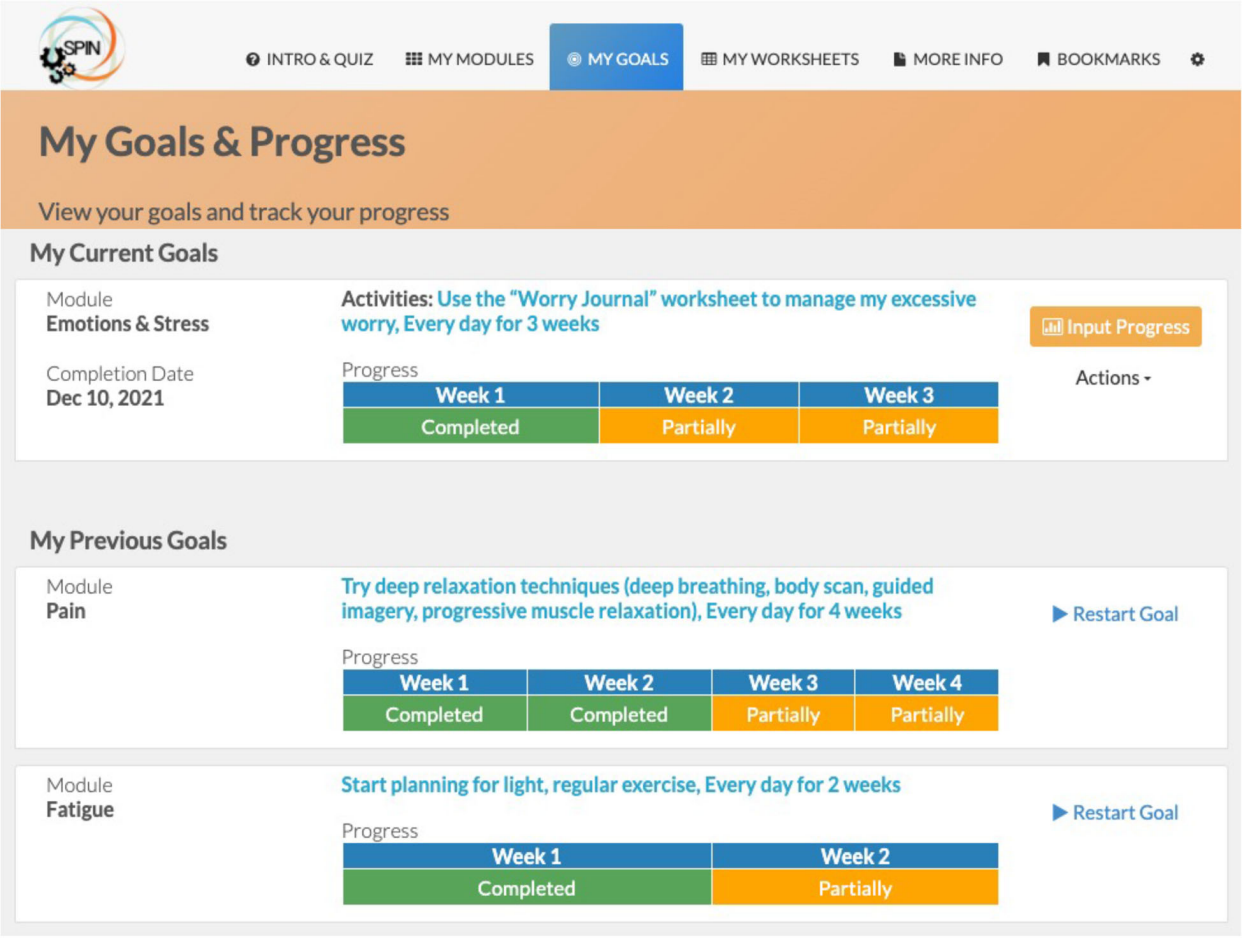
Goals and progress tracking tool

**Fig. 10:**
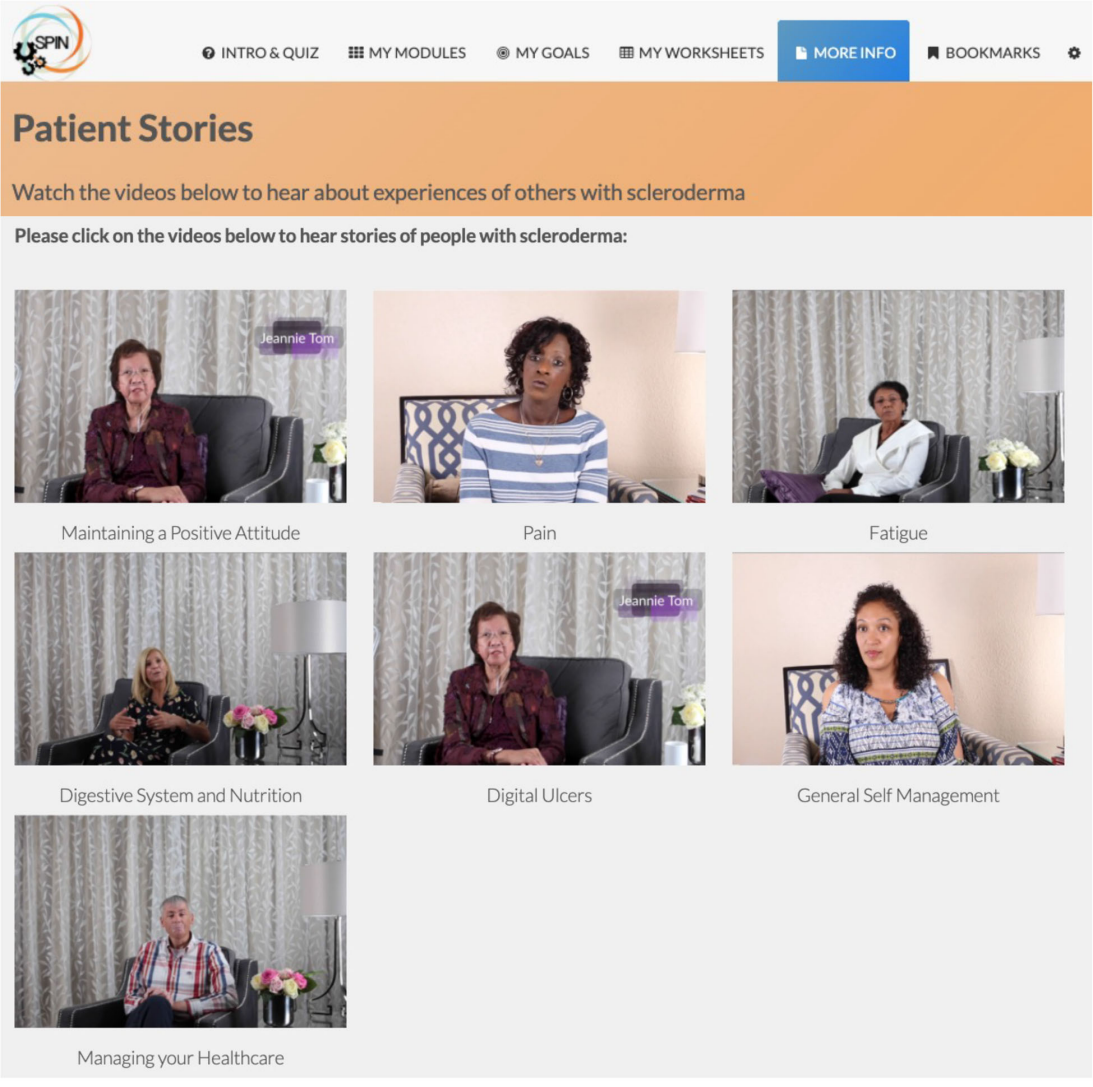
Living with SSc stories page

The SPIN-SELF Program will include access to online program material for the duration of the trial and participation in 8 group videoconference sessions to be delivered over 12 weeks (weekly for 4 weeks then every other week) to support online material. The SPIN-SELF Program curriculum was designed based on key principles of behavior change that have been integrated in successful self-management programs for other chronic diseases [39–42], input from focus groups with people with SSc and health care providers, and SPIN’s Patient Advisory Board. The program follows a similar format as standard self-management programs [11–13, 39] and includes group interaction, along with content modules that focus on self-efficacy enhancing strategies to provide knowledge, skills, and confidence essential to managing physical, emotional, and social consequences of SSc. Each online module includes an educational component, skills teaching components, and goal-setting component. Participants are provided with problem-solving and disease management skills instead of direct solutions to problems. Videos of people with SSc and health care professionals provide instruction on key self-management techniques (see Figs. 3 and 4). The online program’s 9 core content modules focus on (1) coping with pain; (2) skin care, finger ulcers, and Raynaud’s; (3) sleep problems; (4) fatigue; (5) gastrointestinal symptoms; (6) itch; (7) managing emotions and stress; (8) coping with body image concerns due to disfigurement; and (9) effective communication with health care providers (Figs. 5 and 6). In addition to core content modules, the online program includes tools to support goal-setting strategies, goal forms, and worksheets to help patients integrate newly learned skills and techniques into daily routines (Figs. 7, 8, and 9). Social modeling is supported with educational videos of people with SSc who describe their own challenges and their coping strategies (Fig. 10).

Each videoconference-based intervention group will be led by a person with SSc who previously received training as a support group leader via our SPIN-SSLED Program [35, 36]. Group facilitators will support participants to develop self-management skills and integrate educational material from the online toolkit into their daily routines and will moderate group discussions. In addition to their previous training, prior to the trial, each peer facilitator will receive 4 additional training hours divided into 3 sessions to understand their role, including how to promote behavioral change using Specific, Measurable, Attainable, Achievable, Relevant, Time-based (SMART) goals [43–45]; incorporate strategies for communication to encourage participation and support motivation; use active listening skills; model positive feedback; and manage challenging situations.

Each of the 8 intervention sessions will last between 60 and 75 min and include 3 segments: (1) review of modules, including questions and sharing of experiences (20–25 min); (2) education on goal setting, goal achievement, and discussion of goal progress (40–45 min); and (3) presentation of new assignments (5 min). Session 1 will cover an introduction to the SPIN-SELF Program and online modules and tools. Sessions 2–4 will focus on common challenges identified as salient to most people living with SSc (fatigue, sleep, emotions, and stress) to facilitate group learning. Sessions 5 and 6 will provide participants the opportunity to select among other modules most relevant to them (e.g., coping with pain, gastrointestinal symptoms, body image concerns). Session 7 will cover the Effective Communication with Health Care Providers Module. Session 8 involves summarizing progress and planning for the future. Sessions 1 to 4 will be weekly, after which sessions 5 to 8 will be scheduled bi-weekly (8 sessions in 12 weeks in total). See Table 2.

**Table 2:** Outline of SPIN-SELF videoconference group sessions

Session number	Week	Session topic
1	1	• Welcome • Introduction to self-management • Introduction to SPIN-SELF online modules and toolkit • *Assignment:* Review the Fatigue Module
2	2	• Introduce the SPIN-SELF Fatigue module • SMART goal setting (part 1) • Practicing setting SMART goals related to fatigue • *Assignment:* Write a goal on fatigue using the online goal form; review the Sleep Module
3	3	• Introduce the SPIN-SELF Sleep module • SMART goal setting (part 2), discuss progress on fatigue goals • *Assignment:* Write a goal on sleep using the online goal form; practice either fatigue goal or sleep goal; review the Managing Emotions and Stress Module
4	4	• Discussion on the SPIN-SELF Managing Emotions and Stress Module • Check-in on the progress of participants’ SMART goals • Discuss tips on how to overcome common barriers to achieving goals • *Assignment:* Write a goal on managing emotions and stress; continue working on the goal most relevant to you (fatigue or sleep or emotions and stress); participants to select another SPIN-SELF module relevant to them and review it: o Digestive system & Nutrition Module o Itch Module o Coping with Appearance Changes Module o Pain Module o Skin Care Module o Effective Communication with Health Care Providers Module
5	6	• Discuss individually selected SPIN-SELF modules • Check-in on the progress of participants’ SMART goals • Discuss tips on how to stay motivated when working on goals • *Assignment:* continue working on current goals; review another SPIN-SELF module
6	8	• Discuss progress on previously set SMART goals and new goals • Recap on setting SMART goals • Recap on overcoming barriers to achieving goals • Discussion on mindset and habits to be successful in reaching goals • *Assignment:* continue working on current goals; review the SPIN-SELF Effective Communication with Health Care Providers Module
7	10	• Discussion on the Effective Communication with Health Care Providers Module • Discussion on how to prepare for a health care appointment and how to make health care decisions • Check-in on the progress of participants’ SMART goals • *Assignment:* continue working on current goals
8	12	• Discussion on overall progress in self-management goals • Wrap-up of the SPIN-SELF Program and discuss how to continue with the goals after the sessions end

Group sessions will be delivered using the GoToMeeting® videoconferencing platform, which we have used successfully in previous trials [27, 35, 36]. Email and phone support will be available to intervention participants to resolve any problems with the program website or with group session logins. Participants may choose to discontinue their participation in the intervention at any time. We do not envision the need to modify the intervention or intervention assignment for any participants or to discontinue their participation in the program.

All group sessions will be video-recorded, and 25% of the sessions will be randomly selected for intervention fidelity evaluation. Consistent with best-practice recommendations for assessing treatment fidelity [46], this will be done using a checklist that reflects the specific components of each session of the program.

Participants randomized to the usual care control group will receive care as usual but will not have access to the SPIN-SELF Program during the trial period. They will be able to access the online version of the program following completion of all trial assessments, but they will not be able to access the group-based part of the program.

There will be no restriction on any concomitant interventions for participants in the intervention or usual care control groups.

### Trial outcomes and measures

During the feasibility and full-scale portions of the trial, we will assess participants’ experience with the program and will collect outcomes that will be used to evaluate intervention effects in the full-scale portion of the trial. Participant satisfaction with the intervention will be assessed 3 months post-randomization among intervention participants using an adapted version of the 8-item Client Satisfaction Questionnaire (CSQ-8) [47]. During the feasibility portion of the trial, participants will also be asked open-ended questions about their experience in the program, including challenges, positive elements, and suggestions for improvement, using an adapted version of the Patient Education Assessment Tool (PEMAT) [48]. Logins to the online program and usage of program components will also be assessed using automated usage logs collected through the online SPIN-SELF platform. Feasibility elements of trial procedures and the delivery of the SPIN-SELF Program will be tracked by the research team throughout the feasibility trial. See Table 1 for a detailed description of feasibility outcomes.

The primary outcome analysis in the full-scale trial will compare SEMCD Scale scores [38] between participants randomly assigned to the SPIN-SELF Program versus care as usual control at 3 months post-randomization. Secondary outcomes will include SEMCD scores 6 months post-randomization, patient activation scores at 3 and 6 months post-randomization using the Patient Activation Measure (PAM-13) [49], social appearance anxiety scores at 3 and 6 months post-randomization using the Social Appearance Anxiety Scale (SAAS) [50], and functional health outcomes at 3 and 6 months post-randomization using the Patient Reported Outcomes Measurement Information System profile version 2.0 (PROMIS-29v2) [51].

#### CSQ-8 [47]

The 8-item CSQ-8, which measures client satisfaction with health care services, has been adapted to assess participant satisfaction with the SPIN-SELF Program. Items are scored on a 4-point scale from 1 (low satisfaction) to 4 (high satisfaction). Total scores range from 8 to 32, with higher scores indicating higher satisfaction. The CSQ-8 is available in English and French [52].

#### PEMAT [48]

The adapted PEMAT is a 23-item questionnaire that allows participants to provide comments on their experiences with the format and procedures of the trial, the program content, group sessions, and other input. Responses are assessed qualitatively. The PEMAT is available in English and French.

#### Program usage

Usage among participants in the intervention group will be examined via automated intervention usage log data. These data will provide information on online program logins and usage of specific modules.

#### SEMCD [38]

The SEMCD is a 6-item measure that evaluates confidence in ability to manage fatigue, pain, emotional distress, and other symptoms; to do things other than take medications to reduce illness impact; and to carry out tasks and activities that may reduce the need to see a doctor. Items are rated on a 1 (not confident at all) to 10 (totally confident) scale. The total score is the mean of all items, and ranges from 1 to 10. SEMCD scores have been validated in SSc through the SPIN Cohort in English and French [53].

#### PAM-13 [49]

Patient activation, which reflects the extent to which patients effectively engage with and manage their own health [49, 54], will be assessed with the 13-item PAM-13. The PAM-13 includes 13 statements related to participants’ attitudes and perspectives about their health, with four possible responses ranging from (1) strongly disagree to (4) strongly agree, with an additional “Not applicable” option. Using calibration tables, participants’ responses are converted into raw scores ranging from 0 to 100, with higher scores indicating higher activation [55]. PAM-13 scores have demonstrated reliability and validity in many chronic conditions [56–58]. The PAM-13 is available in English and French [55].

#### SAAS [50]

The SAAS consists of 16 items that measure patients’ self-reported anxiety related to situations where one’s appearance will be evaluated, with response options ranging from 1 (“Not at all”) to 5 (“Extremely”). To calculate total scores, the first item (“I feel comfortable with the way I appear to others”) is reverse-coded, all items are summed, and total scores range from 16 to 80, with higher scores indicating greater anxiety. SAAS scores have been validated as a measure of social appearance anxiety for people with SSc through the SPIN Cohort in English and French [59, 60].

#### PROMIS-29v2 [51]

The PROMIS-29v2 measures 8 domains of health status with 4 items for each of 7 domains (physical function, anxiety, depression, fatigue, sleep disturbance, social roles and activities, pain interference) plus a single item for pain intensity. Domain items are scored on a 5-point scale (range 1–5), with different response options for different domains. The single pain intensity item is measured on an 11-point rating scale. Higher scores represent more of the domain being measured; that is, better physical function and ability to participate in social roles and activities, but higher levels of anxiety, depression, fatigue, sleep disturbance, pain interference, and pain intensity. Total raw scores are obtained by summing item scores for each domain, which are converted into T-scores standardized in the general US population (mean = 50, standard deviation = 10). The PROMIS-29v2 scores have been validated in SSc in English and French [61, 62].

### Sample size

A Cochrane Review (*N* = 7,442) of lay-led self-management programs reported a standardized mean difference (SMD) effect size of 0.3 (95% confidence interval [CI] 0.19 to 0.41) for improved self-efficacy in 10 studies [63]. With a 1:1 randomization ratio, for an assumed effect size of SMD = 0.30, two-tailed *α* = 0.05, a mean of 8 participants per group, and intra-class correlation coefficient of 0.08 (based on SPIN-CHAT results) [27], *N* = 471 provides ≥ 80% power. In our recent SPIN-CHAT [27] and SPIN-SSLED trials [35, 36], which used similar group formats, loss to follow-up was well below 10%. Assuming 10% loss to follow-up would require 524 participants. In the 3 months between March 1, 2021 and June 1, 2021, 1007 SPIN Cohort participants completed assessments, of whom 986 completed the SEMCD Scale; of these, 524 (53%) met SPIN-SELF eligibility criteria. Over 50% of SPIN-CHAT [27] participants were recruited externally, and we expect similar external recruitment for SPIN-SELF to supplement SPIN Cohort participants, which will allow us to meet our recruitment target.

### Recruitment

Eligible SPIN Cohort participants identified via routine cohort assessments will receive an email from a SPIN Team member sent using an automated email feature in the *Qualtrics* platform to introduce the SPIN-SELF Program and explain the enrollment procedure and what participation involves. The email will contain a secure *Qualtrics* survey link by which participants will have the opportunity to consent, decline, or request to be contacted by a study team member for more information. Those who consent will also provide days and times when they could take part in group sessions.

Additionally, recruitment announcements will be posted on SPIN’s Facebook page and Twitter account and distributed via SPIN’s patient organization partners in countries with large English-speaking populations (feasibility and full-scale trials; Canada, USA, UK, Australia, New Zealand, Philippines) and French-speaking populations (full-scale trial; Canada, France). Recruitment announcements for non-SPIN Cohort participants will direct potential participants to a different *Qualtrics* survey link with information on the trial and a consent form for the completion of eligibility questionnaires online, items to assess eligibility, similar consent options for trial participation as SPIN Cohort enrollees for those who are eligible, and days and times available for those who are eligible.

Consent forms for the feasibility portion of the trial will explain (1) that the purpose of the feasibility trial is to test the procedures and intervention delivery prior to proceeding to the full-scale trial; (2) that if based on the feasibility trial we determine that no substantive changes are needed to procedures we will proceed directly to the full-scale trial with data from the feasibility study included in the full-scale RCT; and (3) that if substantive changes are required, the feasibility trial data will be analyzed separately from the full-scale trial.

Consent forms for both the feasibility and full-scale portions will explain that (1) new intervention groups will be started on an approximately monthly basis; (2) each month, enrolled participants with availabilities compatible with scheduled groups will be randomly selected and assigned to an intervention group or care as usual control with 1:1 allocation ratio; (3) participants randomized to the SPIN-SELF intervention group, plus those allocated to care as usual, will complete outcome measures online at 3 and 6 months post-randomization through *Qualtrics*; (5) depending on availabilities, it is possible that some enrolled participants will not be able to be randomized; and (6) enrolled participants who do not receive the intervention as part of the trial, either because they are selected for the control group or because they are not randomly assigned to either the intervention group or control, will be offered access to the online-only SPIN-SELF Program version after the full-scale trial but will not be placed into videoconference groups. A member of the SPIN research team will call all consented participants to confirm availability, time zone, and answer any questions they might have prior to including them in the pool of participants eligible for randomization. Potential participants who decline enrolment in the SPIN-SELF trial will not be reassessed for eligibility if they are again eligible at a future SPIN Cohort assessment.

Recruitment will be paused once our enrollment target for the feasibility trial has been reached. We will restart at the beginning of the full-scale trial.

### Random selection and allocation

For the feasibility and full-scale portions of the trial, consented participants will be entered into pools of participants available to participate in the same weekly group based on availability and, for the full-scale trial, language. For the full-scale trial, separate English- and French-language groups will be formed (feasibility only includes English-speaking participants). De-identified codes for participants in each pool will be provided to an external randomization service. Starting with the largest pool, the service will randomly select the largest possible even number of participants (12 to 20 participants to obtain intervention groups of 6 to10 participants) then randomly allocate half to intervention and half to control via single block randomization using R version 3.6.3. For the feasibility trial, depending on scheduling availability and pool sizes, we will form 2–3 pools. For the full-scale trial, we will form as many pools as possible each month until the enrollment target is met.

All participants who are randomized will receive an email to communicate their assignment to the SPIN-SELF Program or usual care. The intervention group email will include the date and time of their first group session and information on how to access SPIN-SELF Program and login to the videoconferencing system. Participants who have not been randomly assigned to either the intervention group or the waitlist will receive an email to inform them that they could not be assigned but will be re-contacted to confirm eligibility and interest and attempted to be randomized when new sessions begin again in a month.

### Blinding and protecting against sources of bias

In most pragmatic trials of education and psychological interventions, including the SPIN-SELF Trial, participants are typically not able to be blinded to intervention status. This may be understood as part of the response to being offered an intervention, as may occur in clinical practice [64]. Another concern relates to the potential for contamination if participants randomized to the control group can also access the program. This risk of this, however, is minimal. Program materials are accessible only via the secure intervention website, and we will request that intervention participants do not share their access information with others. Study staff who interact with participants to provide help with access or other issues will not be blind to intervention status. Outcome assessment will not be blind, since it is self-report and will be done by participants not blind to their status. Statistical analyses cannot be blinded due to the PN-RCT design in which participants in the intervention arm, but not control, are nested.

### Data collection and management

Informed consent for SPIN Cohort and external enrollees will be obtained via the *Qualtrics* survey tool. To ensure accuracy and link to SPIN Cohort data, for SPIN Cohort enrollees an email authentication check will ensure that emails entered match eligible SPIN Cohort participant emails. Data security measures in place at *Qualtrics* are described in the *Qualtrics* security statement [65].

For both the feasibility and full-scale trials, all participants will complete the outcome measures at 3 and 6 months post-randomization through *Qualtrics*. Baseline measures will be completed through regular SPIN Cohort assessments for SPIN participants and through *Qualtrics* for externally recruited participants. The SPIN Cohort uses a secure electronic data management platform designed and managed by the Information Management Services of the Centre for Clinical Epidemiology, Jewish General Hospital, Montreal. All information obtained from participants during the trial will be treated confidentially within the limits of the law. To protect the privacy of participants, a unique participant identification number has been automatically assigned to each participant (SPIN Cohort IDs for Cohort participants and SPIN-SELF IDs for external participants).

Separate from the SPIN Cohort portal, an encrypted database has been created for SPIN interventions, including the SPIN-SELF Program, which includes the usage log information of participants in the intervention group. Participant identification numbers are also available in the usage log to link participants’ intervention and regular cohort data.

During the trial, access to the cohort portal and the encrypted database will be limited to study investigators. Once the trial ends and results are reported, de-identified data will be made available upon reasonable request. No biological specimens will be collected.

### Data analysis

Consistent with the feasibility trial design and small sample size, no hypothesis tests are planned for the feasibility portion of the trial [66, 67]. Instead, we will present a description of the feasibility elements, including participants’ eligibility and recruitment and numbers and percentages of participants who respond to follow-up measures. Use of the internet intervention will be described by presenting the frequency of logins and usage of the specific SPIN-SELF modules. Analysis of outcome measures will include the completeness of data and presence of floor or ceiling effects. Descriptive statistics will be used to provide means and standard deviations for the measures. Qualitative information on participants’ experience using the SPIN-SELF intervention will be used to interpret acceptability related to the group-sessions format, content of the sessions and online program, webpage organization, and navigation. Information related to required resources and management of the program during feasibility will inform any necessary changes to intervention or trial procedures.

In the full-scale trial, we will use intent-to-treat analyses to estimate score differences between intervention and care as usual participants with a linear mixed-effects model fit using the lmer function in lme4 [68]. Score differences and Hedges’ g SMD effect size will be presented with 95% CIs. For all models, to account for clustering in the blocked PN-RCT design, we will fit a random intercept and slope for treatment effect by randomization block and an additional random slope for treatment by intervention group cluster [30, 69]. In main analyses for each outcome, in addition to a fixed effect for assignment to the intervention arm, we will include a fixed effect for baseline score. In adjusted analyses, we will also control for age (years), sex (male vs. female), SSc disease subtype (diffuse vs. limited), disease duration (years since diagnosis), and country (e.g., Canada, France, other vs. USA) as fixed effects.

To minimize the possibility of bias from missing outcome data, we will use multiple imputation by chained equations using the mice [70] package to generate 20 imputed datasets, using 15 cycles per imputed dataset. Variables in the mice procedure will include randomization block, intervention arm, number of videoconference sessions attended, measures of all primary and secondary outcomes at baseline and at 3 and 6 months post-randomization, age, sex, SSc disease subtype, years since diagnosis, country (e.g., Canada, France, UK, Australia, other vs. USA), and race/ethnicity (e.g., Black and other vs. White). Pooled standard errors and associated 95% CIs will be estimated using Rubin’s rules [71].

To estimate average intervention effects among compliers (to be defined pre-analysis based on session attendance or online program usage), we will use an instrumental variable approach to inflate intent-to-treat effects from main models by the inverse probability of compliance among intervention arm participants [72, 73]; 95% CIs will be constructed via a cluster bootstrap approach, resampling at study randomization block and participant levels [74, 75]. For transparency, results for participants with complete data will also be shown.

Participant satisfaction scores for the intervention group will be reported descriptively.

All outcome analyses will be conducted in R (R version 3.6.3; R Studio version 1.2.5042). All analyses will be 2-sided with *α* = 0.05. We will not adjust for multiple analyses since we identified a single primary outcome a priori.

### Trial coordination and data monitoring

The trial will be coordinated by the SPIN Team in Montreal, Canada. The trial will be overseen by the SPIN Steering Committee along with the trial investigators. The Steering Committee will be updated periodically on the progress of the trials. The SPIN Director, together with trial investigators, will be responsible for routine monitoring of data quality and RCT protocol execution. These groups are independent from trials sponsors.

### Risks and potential benefits

We do not anticipate any serious risks or safety concerns associated with participating in the SPIN-SELF Trial. Nonetheless, any reported adverse event will be recorded, and when necessary, the event will be discussed with clinical members within the team and a referral to SPIN’s health care professionals from the recruiting site will be made. Any serious adverse events that occur will also be reported to the Research Ethics Committee.

Although it is hypothesized that the SPIN-SELF Program will improve disease management self-efficacy and functional health outcomes, it cannot be guaranteed that participants will receive any benefits from this study. However, knowledge gained from this study may lead to the implementation and dissemination of the SPIN-SELF Program, which may benefit those living with SSc in the future. There will be no financial compensation for participants in the trial.

### Ethics and dissemination

The SPIN Cohort was approved by the Research Ethics Committee of the Jewish General Hospital, Montreal (#12-123), and by ethics committees of each recruiting site. The SPIN-SELF Feasibility Trial with Progression to Full-scale Trial has been approved by the Research Ethics Committee of the of Centre intégré universitaire de santé et de services sociaux du Centre-Ouest-de-l'Île-de-Montréal (#2021-2777). All participants will provide electronic consent via *Qualtrics* prior to participating in the trial. Any modifications to the protocol which may impact the conduct of the study, including changes of study objectives, study design, patient population, sample sizes, study procedures, or significant administrative aspects will undergo a formal amendment to the protocol. Any such amendment will be submitted to the Research Ethics Committee for approval and documented in the trial’s registration.

The SPIN-SELF Program may improve disease management self-efficacy and health-related quality of life of people with SSc. If effective, the online program will be made available via the SPIN-SHARE online platform [76] and advertised through SPIN’s website, social media platforms, and collaborating patient organizations around the world. Patient organization partners plan to link from their websites to the SPIN-SHARE platform if the program is effective, and plan to deliver the SPIN-SELF group sessions to be led by SPIN-SSLED [35] trained peer-facilitators. Results from the trial will also be disseminated via peer-reviewed journal publication and presentations in national and international conferences. Beyond SSc, the SPIN-SELF program may serve as a model to be adapted for other diseases.

## Trial status

This is the first version of the protocol, finalized on June 10, 2021. Recruitment and enrollment via the SPIN Cohort and social media announcements are planned to begin in August 2021 and will continue until our enrollment target is reached.

## Supplementary Information

**Additional File 1.** Items from the World Health Organization Trial Registration Data Set

**Additional File 2.** Standard Protocol Items: Recommendations for Interventional Trials (SPIRIT) 2013 Checklist: Recommended items to address in a clinical trial protocol and related documents

**Additional File 3.** Participant consent form (English version)

## Data Availability

All data and materials will be provided upon reasonable requests to the corresponding author.

## Abbreviations

CSQ-8: Client Satisfaction Questionnaire-8
cmRCT: Cohort multiple randomized controlled trial
PN-RCT: Partially nested randomized controlled trial
PAM-13: Patient Activation Measure-13
PEMAT: Patient Education Assessment Tool
PROMIS-29: Patient Reported Outcomes Measurement Information System
RCT: Randomized controlled trial
SEMCD: Self-Efficacy for Managing Chronic Disease
SMART goals: Specific, Measurable, Attainable, Achievable, Relevant, Time-based goals
SMD: Standardized mean difference
SPIN: Scleroderma Patient-centered Intervention Network
SPIN-CHAT: SPIN COVID-19 Home-isolation Activities Together
SPIN-HAND: SPIN Hand Function Program
SPIN-SELF: SPIN Self-management Program
SPIN-SSLED: SPIN Scleroderma Support Group Leader Education Program
SPIRIT: Standard Protocol Items: Recommendations for Interventional Trials
SSc: Systemic sclerosis
