# Supplementary material for: The Scleroderma Patient-centered Intervention Network Self-Management (SPIN-SELF) Program: protocol for a two-arm parallel partially nested randomized controlled feasibility trial with progression to full-scale trial
Source: Trials. 2021 Nov 27;22:856. doi: 10.1186/s13063-021-05827-z (PMC8626736; doi:10.1186/s13063-021-05827-z)
Supplement: Supplementary file 1 — Additional File 1. Items from the World Health Organization Trial Registration Data Set [file 13063_2021_5827_MOESM1_ESM.docx]

**Additional File 1.**

| **Data Category** | **Information** |
| --- | --- |
| Primary registry and trial identifying number | ClinicalTrials.gov  NCT04246528 |
| Date of registration in primary registry | Initial Release: January 27, 2020  Last Update: June 1, 2021 |
| Secondary identifying numbers | Unique Protocol ID: 2021-2777 |
| Source(s) of monetary of material support | Lady Davis Institute |
| Primary sponsor | Lady Davis Institute |
| Secondary sponsor(s) | N/A |
| Contact for public queries | Dr. Brett Thombs [brett.thombs@mgill.ca] |
| Contact for scientific queries | Dr. Brett Thombs [brett.thombs@mgill.ca] |
| Public title | The Scleroderma Patient-centered Intervention Network (SPIN) Self-Management Program: Protocol for a Two-arm Parallel Partially Nested Randomized Controlled Feasibility Trial with Progression to Full-Scale Trial |
| Scientific title | The Scleroderma Patient-centered Intervention Network (SPIN) Self-Management Program: Protocol for a Two-arm Parallel Partially Nested Randomized Controlled Feasibility Trial with Progression to Full-Scale Trial |
| Countries of recruitment | Canada, United States, United Kingdom, Australia, New Zealand, Philippines, France |
| Health condition(s) or problem(s) studied | Self-management for participants with systemic sclerosis (scleroderma; SSc) |
| Intervention(s) | SPIN Self-Management Program (SPIN-SELF)  Comparator: usual care |
| Key inclusion and exclusion criteria | Inclusion criteria: Adults ≥18 years old, fluent in English or French; meet 2013 American College of Rheumatology / European League Against Rheumatism SSc classification criteria, have low disease management self-efficacy (Self Efficacy for Managing Chronic Disease Scale ≤ 7.0) |
|  | Exclusion criteria: Participants cannot have been enrolled and assigned to the intervention arm of the initial SPIN-SELF Feasibility Trial |
| Study type | Partially nested randomized controlled trial |
| Date of first enrolment | September 2021 |
| Target sample size | 524 |
| Recruitment status | Not yet recruiting |
| Primary outcome(s) | Disease management self-efficacy (3-months post randomization) |
| Key secondary outcomes | Feasibility trial: Patient satisfaction with the online SPIN-SELF program and program usage |
|  | Feasibility and Full-scale trial: Disease management self-efficacy (6 months post randomization), patient activation, social appearance anxiety and functional health outcomes (physical function, anxiety, depression, fatigue, sleep disturbance, social roles and activities, pain interference) at 3 and 6 months post-randomization |
