# Supplementary material for: The Scleroderma Patient-centered Intervention Network Self-Management (SPIN-SELF) Program: protocol for a two-arm parallel partially nested randomized controlled feasibility trial with progression to full-scale trial
Source: Trials. 2021 Nov 27;22:856. doi: 10.1186/s13063-021-05827-z (PMC8626736; doi:10.1186/s13063-021-05827-z)
Supplement: Supplementary file 3 — Additional File 3. Participant consent form (English version) [file 13063_2021_5827_MOESM3_ESM.docx]

### INFORMATION AND CONSENT TO PARTICIPATE IN

### THE SCLERODERMA PATIENT-CENTERED INTERVENTION NETWORK

SELF-MANAGEMENT (SPIN-SELF) PROGRAM TO IMPROVE DISEASE-MANAGEMENT SELF-EFFICACY IN PATIENTS WITH SCLERODERMA

FEASIBILITY STUDY WITH PROGRESSION TO FULL-SCALE TRIAL

| **Principal Investigator:**  **Funding:** | Dr. Brett D. Thombs  Canadian Institutes of Health Research |
| --- | --- |

**______________________________________________________________________________**

**Introduction**

You are being invited to participate in this study because (1) you are currently a SPIN Cohort participant, and (2) you are eligible based on the inclusion criteria for this study. The purpose of this study is to test SPIN’s scleroderma self-management program (SPIN-SELF Program) procedures and to evaluate if participating in the program improves your confidence in managing your scleroderma. Take as much time as you need to read the following information carefully. Participation in the study is voluntary.

**Purpose of the study**

The SPIN-SELF Program combines access to online modules that focus on problems important to people with scleroderma, along with 8 group videoconference sessions. The topics addressed in the SPIN-SELF Program include (1) coping with pain; (2) skin care, finger ulcers and Raynaud’s; (3) sleep problems; (4) fatigue; (5) gastrointestinal symptoms; (6) itch; (7) managing emotions and stress; (8) coping with body image concerns due to disfigurement; and (9) effective communication with healthcare providers.

During this study we will evaluate our procedures for delivering the program and participant satisfaction with how the group sessions are conducted. If our procedures do not require any major changes, we will also use results from the group sessions to evaluate whether participating in the program improves people’s confidence to manage their scleroderma more effectively. If important changes are required, we will make them and then evaluate the effectiveness of the program only in subsequent groups.

**SPIN-SELF Program**

SPIN-SELF is a brief group videoconference-based intervention that will take place in 8 sessions over 12 weeks. The first 4 sessions are delivered weekly, and the next 4 sessions are delivered every other week. In addition to group sessions, program participants will access and use the online SPIN-SELF Program. Each intervention group will be led by a person with scleroderma who has been trained to facilitate these groups. During the 60-75 minute videoconference sessions, the facilitator will present discussion topics, based on the SPIN-SELF online module topics and will moderate discussion among group participants. The facilitator will support participants to develop self-management skills and to integrate educational material into their daily routines. The objective of the group sessions is to help participants learn skills so they can gain confidence in managing the physical, emotional and social consequences of scleroderma. All sessions will be delivered using the GoToMeeting® videoconferencing platform, which is easy-to-use and has been used successfully in previous SPIN patient programs.

**Study procedures**

If you agree to participate, you can consent electronically at the bottom of this page. Once you have consented, you will be asked to provide your availabilities by selecting time blocks for each day of the week (morning, afternoon, evening). Selection to participate in the trial depends on you having availabilities that match when SPIN-SELF groups will be held. A SPIN Team member will try to reach you within the next 48 hours to answer any questions you may have and confirm your availabilities.

If you would like to talk to a member of the SPIN Team before deciding whether you would like to participate in the study, please indicate at the bottom of the page, and a member of our team will contact you to answer your questions.

**If you are selected to participate in the trial and assigned to a SPIN-SELF group**, you will receive an email informing you of your assigned group, with the date and time of the first session, the topic of the first session, and information on how to login to the videoconferencing system. After your participation in the SPIN-SELF Program, we will ask that you fill out a few study measures. You will complete the study measures twice, one time just after the completion of the program and one time 3 months later. Each time, we will send you an email with a clickable link to the questionnaires. The total time to complete these measures is approximately 15-20 minutes.

**If you are selected to participate in the trial and assigned to a waitlist group**, you **will not** receive the SPIN-SELF Program now, but you will be on a waitlist to receive access to the online modules and toolkit of the program (without group meetings) at the end of the full-scale trial (i.e. once the main study is over). After you are given access to the SPIN-SELF online toolkit and you have a chance to explore it on your own, we will ask that you complete the online study measures in 3 months and in 6 months. The responses you provide are important to the testing of the SPIN-SELF Program. A clickable link to fill out the online measures will be provided in an email. The total time to complete these measures is approximately 10-15 minutes.

It is possible that some enrolled participants will not be able to be randomized to a SPIN-SELF group or the waitlist because there are not enough other participants with similar day and time availabilities. In that case, those participants will be offered access to the online SPIN-SELF toolkit at the end of the full-scale trial. If you enrol but are not able to be selected right away, we will contact you by email to make sure you are still available when we are ready to open new groups.

**Study Measures**

As mentioned above, if you are part of the intervention group you will be asked to fill out online measures at the end of the program and 3 months later. If you are part of the waitlist group, you will be asked to complete online measures 3 months and 6 months after receiving access to the SPIN-SELF online toolkit. These include measures assessing levels of self-efficacy and health-related quality of life. If you are in the assigned to the **intervention group**, you will also be asked to fill out 2 questionnaires about your satisfaction with the program. **To ensure that we are truly assessing the effects of the SPIN-SELF Program, we ask that you do not share any of the program materials with other people with scleroderma until we let you know that the trial has been completed.**

**Potential risks and benefits of participation**

This study is of minimal risk. We do not anticipate that there will be any adverse events from participating in the SPIN-SELF Program. We cannot guarantee that you will receive any benefits from this study. However, information learned from this research may lead to better interventions to target important problems for people living with scleroderma, which may benefit other patients in the future. It is possible that using the SPIN-SELF Program may improve your self-management efficacy, but this has not been proven.

**Voluntary participation/withdrawal**

Your participation in the study is voluntary. You have the right to refuse to participate or to withdraw from the study, without giving any reason, at any time, and without any prejudice to you.

In the event that you withdraw, or if you need to be withdrawn from the study, all information collected up until that point for the purpose of this study may be used in order to preserve the scientific integrity of the study.

**Confidentiality**

All information obtained about you during this study will be treated confidentially within the limits of the law. During your participation in this study, the researchers responsible for this project and their staff will audio and video-record the SPIN-SELF group sessions. The sessions will be recorded for the purpose of review and training, and all identifying information will be kept confidential. At the start of each group session, facilitators will remind group participants that in order to maintain the integrity of the study and to protect the confidentiality of these sessions and image rights of participants, all information shared during the group session should not to be shared outside of the group, SPIN-SELF Program materials are confidential and should not be shared outside the group, and participants should not take screenshots or personal recordings of the group sessions. The session recordings will be uploaded to a private password protected album on Vimeo for participants to review should they miss a session. Only the study team members and members of your assigned group will have access to the session recordings.

Recorded training sessions and associated information gathered will be kept for 10 years by the researchers responsible for the study. To protect your privacy, you will be assigned a unique participant identification number within the study data system, to make sure your data is not associated directly with your name. Your data will be kept in an encrypted database. Only requests authorized by the principal investigator (Dr. Brett Thombs) will be granted access to this encrypted information. The recordings of the sessions will be destroyed by eliminating the digital files. Recordings will be kept at the Jewish General Hospital under the responsibility of Dr. Brett Thombs. The Research Ethics Board of the CIUSSS-West Central Montreal may look at research files for the purpose of monitoring this research.

Some of the data, including your responses to study questionnaires, will be kept on the servers of the company Qualtrics. Data security measures in place at Qualtrics are described in the Qualtrics security statement (http://www.qualtrics.com/security-statement/).

A summary of anonymized study data may be published or shared with others in scientific discussions. We will not include your name or any information that could be used to identify you in any scientific publications or communications that will follow this study.

**Contact for further information**

Thank you for taking the time to read the information about this study. If you have any questions or concerns now or at any time about the SPIN-SELF feasibility study with progression to full-scale trial, you may contact the SPIN-SELF team at [spinself@gmail.com](mailto:spinself@gmail.com) or at [1-800-370-5099](tel:1-800-370-5099) (Canada and USA toll-free).

If you have any questions or concerns regarding your safety and rights as a participant in the feasibility study with progression to full-scale trial or if you would like to discuss your participation within the SPIN-SELF study with an individual not directly involved in the research, we invite you to call Rosemary Steinberg, Local Commissioner of Complaints & Quality of Services, Jewish General Hospital, at (514-340-8222, ext. 25833).

**I have read the consent form and understand the terms of the SPIN-SELF Feasibility Study with Progression to Full-scale Trial. I agree to participate. I further agree that the data collected in the feasibility study may be used in the full-scale trial.**

- - After consenting, continue to the availabilities survey.
  - Please enter your email address (the one you use to log into the SPIN Cohort) below to confirm your choice: _____________________
  - Please provide us with your phone number linked to your SPIN ID so members of the research team can contact you about the study:
    - Phone number (10 digits no spaces or dashes): _____________________
    - Time zone: __________________
    - Country: __________________Preferred time to be contacted: (morning, afternoon, evening)

**I have read the consent form and I would like to ask questions to a member of the SPIN research team before I decide whether or not to participate:**

- - Please select your preferred contact method:
  - Phone call (if selected, the following message appears “Please provide your phone number and contact details linked to your SPIN ID so a member our team can contact you about the study” with the following options displayed):
    - - Phone number (10 digits no spaces or dashes): _______________
      - Time zone: __________________
      - Country: __________________
      - Preferred time to be contacted: (morning, afternoon, evening)
  - Email (if selected, the following message appears “Please provide your email address linked to your SPIN ID so a member our team can contact you about the study”)
    - - Email address: _____________________

**I do not consent to participate in the SPIN- SELF Feasibility Study with Progression to the Full-scale Trial.**

- **Optional: Would you be willing to provide us feedback?** (this message appears if this option is selected with a text box): In the text box below, please let us know (1) The reason(s) why you are declining to participate in the study; (2) Any recommendations you may have for improvements that would make you more willing to participate. Your feedback could help us improve our study design and procedures.
